# The Potential of ACTH in the Genesis of Primary Aldosteronism

**DOI:** 10.3389/fendo.2016.00040

**Published:** 2016-05-23

**Authors:** John W. Funder

**Affiliations:** ^1^Hudson Institute of Medical Research and Monash University, Clayton, VIC, Australia

**Keywords:** aldosterone, ACTH, cardiovascular, cortisol, circadian rhythm

## Abstract

Aldosterone is a homeostatic hormone, rising in volume depletion, sodium deficiency, and potassium loading, in response to angiotensin11 and elevation of plasma potassium. Pathophysiologically, in primary aldosteronism (PA) aldosterone levels are inappropriate for the patient’s sodium and potassium status, and thus outside the normal feedback loop. ACTH is equivalent with A11 and [K^+^] in elevating aldosterone: its effects differ from those of the other secretagogues in four ways. First, it is not sustained; second, it raises aldosterone and cortisol secretion with equal potency; third, it is outside the normal feedback loops, reflecting the epithelial action of aldosterone; and finally its possible role in driving inappropriate aldosterone secretion (aka PA) is not widely recognized. Thirty years ago, it was shown that on a fixed sodium intake of 175 meq/day 36 of 100 unselected hypertensives, in whom PA has been excluded on contemporary criteria, had 24 h urinary aldosterone levels above the upper limit of normotensive controls. More recently, the dexamethasone enhanced fludrocortisone suppression test (FDST) showed 29% of unselected hypertensives to have plasma aldosterone concentrations above the upper limit of normotensive controls. In subjects negative for PA on the FDST, 27% were extremely hyper-responsive to ultra-low dose ACTH infusion; the remaining 73% showed minimal aldosterone elevation, as did normotensive controls: all three groups had negligible cortisol responses. On treadmill testing, no differences were found between groups in (minimally altered) ACTH and cortisol levels: hyper-responders to ultra-low ACTH, however, showed a major elevation in PAC. The implications of these studies, when validated, are substantial for PA, in that approximately half of hypertensive patients appear to show inappropriate aldosterone levels for their sodium status. The physiological role(s) of ACTH as an acute aldosterone secretagogue, and the mechanisms whereby its continuous secretion is curtailed, remain to be established.

## Background

For many years, most studies on aldosterone have focused on its action on renal salt and water transport, undeniably its major homeostatic role. More recently, following the RALES trial of the effects of spironolactone in progressive cardiac failure (1), a range of extraepithelial effects, primarily cardiovascular, has been shown in animal studies (2–4). The doses of aldosterone (or DOCA) used, and the extent of salt loading, make extrapolation to physiology difficult, and to human pathophysiology doubly difficult. That said, PA clearly has a higher cardiovascular risk profile than age-, sex-, and blood pressure-matched essential hypertension (EH) (5, 6): whether this is a primary effect of aldosterone on the heart and/or blood vessels, or secondary to the increased total body sodium, is yet to be established (7).

The secretory response to what are commonly regarded as its principal regulators (angiotensin II, plasma potassium concentration) is prolonged as long as the stimulus persists. In contrast, ACTH elevates aldosterone acutely but transiently. At least two features of this response are unclear. The first is the organ(s) and processes targeted by the acute elevation; the second the mechanism(s) whereby this acute response is curtailed. Given that the circadian rhythm of aldosterone secretion clearly precedes that of cortisol, both steroids may have roles as “Zeitgebers,” effecting diurnal changes in their respective physiologic target tissues. In addition to its circadian variation, however, ACTH is elevated by stress, physical, or psychological. Whereas stress responses to elevated cortisol have been widely studied, those in response to elevated aldosterone appear to have escaped attention. It may be that they form part of the classical “fight or flight” response, by acutely (non-genomically) activating mineralocorticoid receptors (MR) in the vasculature, a physiologic aldosterone target tissue expressing the specificity-conferring enzyme 11β-hydroxysteroid dehydrogenase type 2 (11β-HSD2) (8). A similar rapid vasoconstrictor response follows the acute aldosterone elevation upon orthostasis.

What curtails the elevation of aldosterone secretion despite continued high ACTH levels is currently unclear. One attractive possibility is that it reflects a genomic effect of maintained high levels of glucocorticoid secretion. Heterozygous glucocorticoid receptor (GR) (±) null mice show persistently elevated levels of both corticosterone and aldosterone levels, consistent with elevated levels of ACTH and impaired glucocorticoid negative feedback on aldosterone secretion (9). To date, there appear to be no reports on levels of aldosterone in patients with hypercortisolism due to GR abnormalities leading to increased ACTH levels. There are, of course, additional/other mechanisms whereby the acute elevation of aldosterone in response to ACTH might be curtailed.

Finally, it is accepted that under normal circumstances, ACTH acts with equivalent potency as a secretagogue for cortisol and aldosterone. Recently, as will be discussed in detail below, there is evidence that aldosterone secretion appears supersensitive to very low dose ACTH at levels which do not elevate cortisol secretion (10).

## Primary Aldosteronism

The first description of the role of ACTH came over 50 years ago, with the description by Sutherland et al. of a patient with “hypertension, increased aldosterone secretion and low plasma renin activity relieved by dexamethasone” (11). This constellation, variously termed glucocorticoid-suppressive hyperaldosteronism (GSH) or glucocorticoid-remediable aldosteronism (GRA) is now termed familial hyperaldosteronism Type-1, and its genesis demonstrated in an early paper from the Lifton laboratory (12). This heritable condition reflects a crossover event during ancestral meiosis, wherein a chimeric gene with the 5′ end of CYP11 B1 (11β hydroxylase) and the 3′ end of CYP11 B2 (aldosterone synthase) are fused. This results in a sequence, which is expressed throughout the zona fasciculata, producing high levels of aldosterone in response to normal levels of ACTH, rather than the normally much lower levels from aldosterone producing cell clusters (13) in the zona glomerulosa. Suppression of ACTH secretion by dexamethasone lowers aldosterone levels and replaces (more or less) the secretion of endogenous glucocorticoid.

Arguably, the first of the studies directly addressing the potential role of ACTH in PA was by Kem et al. (14). Seven PA patients [three ideopathic adrenal hyperplasia (IAH) and four aldosterone producing adenoma (APA)] were studied, with the conclusion that “these data indicate that ACTH frequently is the dominant stimulus of the episodic secretion of aldosterone in patients with either adrenal adenomas or hyperplasia.” Couple this with a report 4 years later by Helber et al. (15) entitled “Evidence for a subgroup of essential hypertensives with non-suppressible excretion of aldosterone during sodium loading.” The effect of sodium loading (175 μg/day for 6 days) on 24 h urinary aldosterone (UA) was determined in three groups – 56 healthy controls, 100 patients with EH, and 16 patients with PA (12 APA, 4 IAH). In healthy controls, the upper limit of UA was <6 μg/day; all the PA patients were unsuppressed (IAH 8–20, APA 23–64 μg/day); 64 of the EH patients suppressed UA to levels in the normal range; but 36 did not, with values above 6 and up to 16 μg/day. Non-suppressing EH patients showed a mean UA value of 10 ± 3 (SD) μg/day; those who suppressed a mean of 2.7 ± 1.4 (SD). Plasma [K^+^] in the 36 non-suppressors was 3.81 ± 0.44 meq/L and in the 64 EH suppressors was 4.26 ± 0.37 meq/L (*p* < 0.001). Finally, in response to 4 weeks on 200 mg/day spironolactone, blood pressure fell by 22 mmHg in non-suppressors, 21 mmHg in the PA patients, and 9 mmHg in the 64 EH patients who suppressed. The authors conclude, “We believe, therefore, that our patient groups with inadequate high aldosterone suppression during Na^+^ loading may reflect qualitatively a similar pathogenetic defect in mineralocorticoid secretion as “hyperplasia patients” with hypermineralocorticoidism.”

These data, based on 24 h urinary rather than spot plasma aldosterone, remained unremarked for more than three decades. By the use of variously stringent cut-offs in both the screening test for aldosterone [the aldosterone to renin ratio (ARR)] followed by confirmatory testing, PA is currently gazetted to affect 5–13% of referred hypertensives. Add the 16 confirmed PA to the 36 non-suppressors found by Helber et al. in patients referred to the University of Cologne, and the figure stands at 45%, which would currently be regarded as totally out of court, given the understandable focus on the most florid expression of PA, sometimes dubbed “low hanging fruit.”

Where this at face value improbable figure starts to make sense is the incorporation of ACTH status into confirmatory/exclusion testing. Currently, any one of half a dozen procedures are used around the world, of which the fludrocortisone-saline suppression test (FST) is sometimes regarded as the “gold standard” – 4 days of fludrocortisone, sodium, potassium supplementation, and often (but not always) hospitalization. In a series of papers from the Piaditis group in Athens (10, 16–19), the potentially confounding effect of ACTH on values obtained in the FST has been addressed in two ways. First is the inclusion of 2 mg dexamethasone at midnight on the last day of testing [fludrocortisone suppression test (FDST)], with initial results that were arresting, in both a group of control normotensives with normal adrenals on imaging, and a second group of essentially unselected hypertensives (16).

On the FDST, the 97.5% upper limit of normal for plasma aldosterone concentration (PAC) was found to be 74 pmol/L, and for ARR 32 pmol/mL, in the 72 normotensive controls. Among the 180 hypertensives, 56 (31%) showed upright PAC values above 74 pmol/L, combined with an ARR >32 pmol/mU on day 5, evidence for a much higher percentage of unselected hypertensives having inappropriate aldosterone secretion, i.e., PA. In prior studies from the same group, the classical saline infusion test (SIT) was compared with a post-dexamethasone SIT; the latter showed a similar (24%) prevalence of PA, more than double the 11% found on the commonly used SIT (17). More recently, much larger groups of controls (*n* = 100) and hypertensives (327) have been studied, with results essentially identical to the initial study, and a prevalence of PA of 29% (18). A recent overview by Piaditis and colleagues (19) compares these latter figures for prevalence with those from other centers absent dexamethasone loading, which range from 1 to 18%.

There are a number of factors that may conspire toward explaining the difference with and without dexamethasone. The first is the reliance on plasma cortisol as a surrogate for ACTH: just as their circadian secretion appears different, the responses to stress (see below) may differ. Second is the single make-or-break value for PAC, close to the nadir of circadian levels, on which both screening (ARR, PAC above some arbitrary cut-off) depend. It is just possible that Helber et al. got it right, and that UA integrates secretion over 24 h, which may be more pathophysiologically relevant. Finally, it may be that in the historically nephrocentric universe of stimuli to aldosterone secretion angiotensin II reigns supreme, plasma [K^+^] admitted almost as an afterthought, and a continuous role of ACTH almost entirely neglected. We may have to extend the sentence quoted earlier from Kem et al. “These data indicate that ACTH frequently is the dominant stimulus of the episodic secretion of aldosterone in patients with either adrenal adenomas or hyperplasia” (14) from the pathophysiological to the realm of physiology, in control normotensives [to halve the 97.5% cut-off in the FST (6 meq/L) to 3 meq/L in the FDST], and (incidentally) in only ~70% of essential hypertensives.

There would also appear to be another dimension in which ACTH may be a probable actor in inappropriate hypersecretion of aldosterone. The data also come from the Athens group, and address patients with EH who previously tested negative for PA by the FDST. In this study (10), 113 of such hypertensives and 61 normotensive controls underwent an ultra-low dose ACTH-stimulation test, followed by a treadmill test. On the basis of the cut-off values for aldosterone (47 ng/dL) and ARR (2.7 ng/dL/μU/mL) at 15- or 30-min post ACTH (97.5% percentile value in controls), 30/113 (27%) of hypertensives recorded values for both PAC and ARR above the cut-off (HYPER group), and 83/113 (73%) did not (EH: ESHT group). Mean values for aldosterone post-ACTH were identical in the control and ESHT groups, and approximately threefold higher at both 15 and 30 min in the HYPER group; cortisol levels post-ACTH were the same in all three groups.

The authors then extended their studies from testing the adrenal responses to ultra-low ACTH to real life (more or less), with all participants undergoing a treadmill stress test to 80% of age-adjusted maximal cardiac pulse, 24 h after the ultra-low ACTH test in each project. When the results of both tests were aggregated and analysed, the 30 patients in the HYPER group showed a twofold to threefold greater elevation over baseline compared with controls or ESHT: no group showed an elevation in plasma cortisol at peak or recovery.

These studies are prismatic, but not perfect. In addition to issues canvassed in the accompanying editorial (20), other questions might be raised. Variances for all groups in terms of response to ultra-low ACTH are extraordinarily low, even when expressed as they are at SEM; this is much less the case for aldosterone responses to treadmill testing, but those for the other two groups remain tight. In contrast, the variances in terms of cortisol response are enormous. The FDST test was done on days 4–7, after ultra-low ACTH on day 2 and the treadmill on day 3. While this may have been convenient, it presumably excluded an unspecified number of subjects who had undergone the two previous tests. There would thus appear to be a corpus of data on such subjects, in addition to the 113 hypertensives who “failed” the FDST. From this perspective, these patients may have been of more interest than the adventitious KCNJ5 data.

These questions said and done, the data from these studies (10, 16–19) taken together argue powerfully that we need to reconsider our currently accepted values for the prevalence of inappropriate aldosterone secretion (aka PA). The current figures of 1–18% of hypertension – on the basis of strict or relaxed cut-offs, referred or general practice patients – are testimony to diagnostic frailty rather than variation in patient populations: what the first three of the Athens studies have shown is a potential confounder in unrecognized roles for ACTH in testing. The implication of the three studies (16–18) is that ~ 30% of unselected hypertensives have autonomous aldosterone secretion independent of the classic secretagogues, a much higher figure than is currently acknowledged.

The ultra-low ACTH/treadmill studies open a quite distinct scenario, that of heightened zona glomerulosa sensitivity to everyday stresses, accounting for a similar fraction of hypertensive subjects. Just as the driver of PA is unclear in the majority of PA diagnosed post-FDST, so it is for the genesis of the adrenal sensitivity. What needs to be done is to validate the generalizability of past findings, not by slavishly repeating the present protocols, given the difficulty in persuading control subjects to undergo imaging and FDST, but by doing a comparison of the seated saline suppression test (SSST) with and without 2 mg dexamethasone at midnight on the day before (FSSST). In preliminary studies, Michael Stowasser’s laboratory has shown excellent comparability between the SSST and the FST (21). Subjects negative on the FSSST might then be enrolled in an ultra-low dose ACTH study, including if possible a 24 h urine to measure integrated aldosterone secretion, in addition to values obtained during the test.

If, as I anticipate, this series of findings from Athens can be generalized, we need to recognize that PA affects not ~ 10% of hypertensives but over half. Currently, major interest and considerate resources are devoted to seeking, for example, additional somatic mutations possibly involved in oversecretion of aldosterone from APA: it may be much less fun grinding through clinical studies, but the outcome for hypertensive patients is likely to be far greater. If over half of hypertensive patients have PA, and fewer than 1% are even screened let alone diagnosed and treated in any jurisdiction, the case for inclusion of low dose MR antagonist in first line antihypertensive therapy would appear to be incontrovertible.

## Author Contributions

The author confirms being the sole contributor of this work and approved it for publication.

## Conflict of Interest Statement

The author declares that the research was conducted in the absence of any commercial or financial relationships that could be construed as a potential conflict of interest.
